# Pitfalls in the diagnosis of eating disorders: a case series from infancy to adolescence

**DOI:** 10.1007/s40519-025-01781-w

**Published:** 2025-08-28

**Authors:** Adele Fiordelisi, Micol Stivala, Roberta Pellegrino, Elisabetta Innocenti, Sandra Trapani

**Affiliations:** 1 Pediatric Unit, Meyer Children’s Hospital IRCCS, Viale Pieraccini 24, 50139 Florence, Italy; 2 Neuroscience Department, Meyer Children’s Hospital IRCCS, 50139 Florence, Italy; 3 Department of Health Sciences, University of Florence, 50139 Florence, Italy

**Keywords:** Eating disorders, Anorexia nervosa, Acute feeding disorders of infancy, Medical mimicker

## Abstract

**Purpose:**

Eating disorders, encompassing a spectrum of conditions, significantly affect individuals throughout their lifespan, especially children and adolescents. Because of their rising incidence, misdiagnosis remains a challenge in paediatrics.

**Methods:**

This study presents an educational case series documenting organic disorders that were initially misdiagnosed as feeding and eating disorders.

**Results:**

Through comprehensive assessments, including medical history, physical examinations, and specialized evaluations, we stress the importance of ruling out medical conditions that can mimic an eating disorder at presentation. Timely recognition and referral to specialized care are essential for improved outcomes.

**Conclusion:**

We highlight the crucial role of continuous reassessment, particularly in cases of evolving symptoms or inadequate response to treatments. A multidisciplinary approach in diagnosing and managing paediatric feeding and eating disorders is of fundamental importance to ensure prompt identification and adequate referral.

*Level of evidence* IV.

## Background

Eating disorders are serious and potentially life-threatening illnesses that impact individuals across their lifespan, particularly affecting the physical and psychological development of children and adolescents [1]. The fifth edition of the Diagnostic and Statistical Manual of Mental Disorders (DSM-5) has changed the terminology “eating disorders” to “Feeding and Eating Disorders” mentioning eight categories: anorexia nervosa, bulimia nervosa, and avoidant-restrictive food intake disorder are the most frequent [1]. Eating disorders are a major cause of mortality and morbidity among adolescents, being the leading cause of death among females between 5 and 24 years of age [2]. Paediatricians should recognize the catastrophic implications of eating disorders on patients’ lives and the heterogeneous presentation of such conditions. Their timely recognition and prompt referral to specialist care are crucial for the achievement of favourable outcomes. However, presenting features of low weight, decreased appetite, fatigue, and unusual or avoiding eating habits are a common reason for seeking medical attention, especially in children and adolescents, even in some other medical conditions. In this case series, we documented several paradigmatic clinical cases initially misdiagnosed as feeding and eating disorders.

### Case 1

A 3-month-old breastfed girl presented to her primary care paediatrician for poor weight gain after her second month of life. Formula feeding was introduced; however, each meal never exceeded 50–60 g, with the infant refusing to consume adequate amounts of milk. Due to persistent underfeeding, the infant was evaluated at a peripheral hospital where blood tests (including gas analysis, ammonia, urine analysis and culture) and instrumental exams (abdominal ultrasound scan (US)), were unremarkable. Second-level investigations (lipase, amylase, sweat test, blood and urine amino acids, acylcarnitine, and urinary CMV analysis) also yielded negative results. Because of symptoms persistence, she was referred to our tertiary-level hospital. The patient was assessed by our gastroenterologist, who first hypothesized an infant feeding disorder and initiated a supportive programme including a speech therapist, a dietitian, and a psychologist. However, to complete the diagnostic work-up, despite an unremarkable neurological physical examination, a transfontanellar US was performed, revealing a homogeneously echogenic signal at the supra-chiasmatic space. This finding prompted second-level imaging with a brain magnetic resonance imaging (MRI) which showed a polylobate expansive lesion above the sella at the hypothalamic-chiasmatic level; the histological examination of a biopsy specimen revealed a hypothalamic-chiasmatic astrocytoma.

### Case 2

A 15-year-old girl presented to our hospital due to a weight loss of 12 kg over 8 months, associated with abdominal pain, decreased appetite, and social isolation. Over time, her condition had deteriorated, with worsening of abdominal pain, eating refusal, and post-prandial vomiting. Additionally, she experienced social withdrawal and avoided school, reporting difficulty maintaining a seated posture. The diagnostic suspicion upon admission was an eating disorder combined with a depressive mood disorder. The girl was then referred to a psychiatric consultation, and antidepressant treatment was started without any improvement. Therefore, she was admitted to our Paediatric Unit. At admission, physical evaluation revealed right gluteal painful swelling and erythema. Laboratory tests showed elevated CRP (20 mg/dL) and erythrocyte sedimentation rate (ESR) (87 mm/h) and hypoalbuminemia (3.3 g/dL). The intestinal US demonstrated diffuse colonic wall thickening with increased vascularization. Further investigations for suspected inflammatory bowel disease (IBD) included a pelvic MRI, which revealed a complex perianal disease. A complete endoscopic assessment revealed ileocolonic mucosal inflammation consistent with Crohn’s disease (CD), subsequently confirmed by the pathology report.

### Case 3

A 14-year-old boy, affected by autism spectrum disorder, was admitted to our ED due to weight loss (− 7 kg in the past year), recurrent abdominal pain, and diarrhoea. A year prior to symptoms onset, he began an elimination diet, coinciding with changes in his bowel habits, alternating constipation and diarrhoea, occasionally accompanied by blood in the stools. The primary care paediatrician identified perianal fissures and referred the patient for a dietary assessment, suspecting an eating disorder. In the subsequent months, an increased rate of blood in the stools and worsening diarrhoea prompted his admission to our hospital’s ED and then to our Paediatric Unit to rule out an IBD. On admission, blood tests revealed iron deficiency anaemia with negative inflammatory markers. Dietary evaluation confirmed extremely selective eating habits, although the daily calorie intake was within the age-appropriate range. Due to the persistence of recurrent abdominal pain, bloody diarrhoea, and weight loss, a complete endoscopic assessment revealed a polyp carpet throughout the whole colon, along with some polyps in the gastric fundus. Histopathological and genetic examination confirmed a de novo presentation of familial adenomatous polyposis.

### Case 4

A 14-year-old girl who presented to our hospital with a one-month history of increased anorexia, fatigue, vomiting, and progressive weight loss (11 kg in one year). The patient reported a metallic taste in her mouth, nausea, and vomiting. Additionally, she complained of fatigue impacting daily life activities, including eating. Her symptoms progressively worsened, leading also to a significant reduction in daily fluid intake (200–250 mL). For the abovementioned symptoms, the patient was referred by her primary care physician to an eating disorders centre, and she was admitted to the Psychiatry Ward. The initial psychological assessment showed eating restriction since age 11, coinciding with her father’s diabetes diagnosis. The girl feared developing diabetes by consuming excessive food. Initial blood tests showed mild anaemia and a slight elevation in serum creatinine and blood urea nitrogen (BUN), initially attributed to moderate dehydration and malnutrition. However, her symptoms worsened despite psychotherapy, rehydration, and enteral nutrition via nasogastric tube, and her renal function significantly deteriorated, prompting transfer to our Paediatric Unit. On admission, right conjunctival hyperemia was observed and the patient complained of photophobia. Blood tests confirmed moderate renal insufficiency with estimated glomerular filtration rate of 32.6 mL/min/1.73m^2^ according to Schwartz [3], proteinuria (28 mg/kg/24 h), glycosuria (420 mg/dL) with normal blood glucose. The ophthalmologic evaluation revealed anterior uveitis of the right eye. Further investigations, including kidney biopsy, led to the diagnosis of tubulointerstitial nephritis and uveitis (TINU) syndrome.

### Case 5

A 16-year-old girl was admitted to our ED presenting with fatigue, post-prandial vomiting, and progressive weight loss of 8 kg over the last two months. Family history revealed psychiatric disorders in both parents, including eating disorders. Physical examination revealed poor general conditions and severe malnutrition (BMI 12.6 kg/m^2^); the remaining physical exam was otherwise unremarkable. Blood tests showed mild microcytic anaemia. The suspicion of an eating disorder prompted a child neuropsychiatric evaluation, which revealed mood deflection and emotional lability. The patient was then admitted to the Neuropsychiatry Department, where psycho-diagnostic tests confirmed the diagnosis of anorexia nervosa-restricting type. Enteral feeding and treatment with antipsychotic drugs were started. However, the worsening of anaemia despite oral supplementation prompted a transfer to our Paediatrics Unit. She was monitored through faecal occult blood test (FOBT), which yielded positive results. The patient’s condition and anaemia further worsened, requiring red blood cell transfusion. Concomitantly, the patient presented melena. Therefore, she underwent an upper gastrointestinal (GI) endoscopy showing a whitish and easily bleeding polypoid formation of the paracardial area, subsequently diagnosed as a HER2-positive gastric adenocarcinoma by histological examination.

### Case 6

A 7-year-old girl presented to our ED with a 4-week history of weight loss and lower limb oedema, subsequently extended to the abdomen and eyelids. Her medical history included type I jejunal atresia, neonatal giant cell hepatitis, intestinal malrotation, gallbladder agenesis, ventricular septal defect, and short stature. Although there was no distortion of body image or voluntary caloric restriction, her history included sporadic vomiting episodes and trichotillomania. The primary care paediatrician had referred her to a neuropsychiatric consultation. Upon admission, she was moderately malnourished. Physical examination revealed tenderness in the abdomen with generalized oedema. Blood tests showed microcytic hypochromic anaemia and severe hypoalbuminemia. The abdominal US revealed ascites and hypoperistaltic small bowel loops. The oral contrast study showed dilated small bowel loops and distension of the distal ileum with slow transit. Upper GI endoscopy identified a big trichobezoar causing stenosis in the distal duodenum and jejunum. The patient underwent laparotomy for the removal of a large trichobezoar (measuring 8 × 6 cm) through an enterotomy, followed by remodelling of a dilated jejunum loop.

### Case 7

A 12-year-old boy was admitted to our ED for a 6-month history of food refusal, vomiting, marked asthenia, progressive weight loss, and behavioural difficulties. Furthermore, oppositional and anxious-depressive traits with extremely selective eating emerged from his history. Upon admission, the boy appeared malnourished (BMI 12 kg/m^2^) and dehydrated. The blood tests showed mild hyponatremia with normal potassium levels, leucocytosis with negative inflammatory markers, and minimal hypoalbuminemia. The instrumental investigations, including small bowel X-ray, upper GI endoscopy, and brain MRI, were all negative. During hospitalization, the patient was fed by parenteral and then by enteral nutrition via nasogastric tube with a suboptimal weight increase. Psychopathological evaluation revealed oppositional defiant disorder. Due to negative etiological examinations and significant psychopathology, the patient was transferred to the Child Neuropsychiatric Unit with suspected eating disorder. Monitoring blood tests documented persistent hyponatremia with potassium levels at the upper limits. Blood pressure measurements showed values above the upper normal limits. Therefore, an endocrinological assessment was performed: cortisol level was markedly reduced and corticotropin level increased. Contrast abdominal computerized tomography (CT) showed poor representation of the adrenal glands. Autoantibody testing revealed positivity for anti-adrenal antibodies, allowing the diagnosis of autoimmune adrenal insufficiency (Addison’s disease).

## Discussion

The first case exemplifies the need to rule out serious medical conditions during differential diagnosis. Diencephalic syndrome is a rare but potentially fatal syndrome caused by a tumour in the diencephalic region. In infants with failure to thrive, a thorough neurological physical examination is mandatory, ideally substantiated by a transfontanellar US. On the other hand, the application of clinical scores such as Wolfson criteria may be helpful [4]. These have been validated as a screening tool for infants with poor feeding or food refusal and include young age (< 2 years), some pathological feeding patterns, and anticipatory gagging. Interestingly, patient 1 did not meet such criteria, underlining their high sensitivity. Therefore, when facing an infant refusing to eat, paediatricians should also consider congenital intracranial tumours which are severe and life-threatening conditions, although very rare; infantile brain tumours constitute approximately 10% of all paediatric central nervous system tumours often occurring in the first six months of life [5].

Another differential diagnosis of feeding and eating disorders is represented by IBDs, especially CD [6, 7]. Failure to thrive, weight loss, and GI symptoms are usual hallmarks of both eating disorders and IBDs. A thorough medical history should investigate the occurrence of diarrhoea and blood in the stools, although not always present, as highlighted in case #2. Physical examination should include the careful evaluation of abdominal masses and tenderness, and of the perianal region. In suspected cases, serum inflammatory markers and faecal calprotectin should be tested. Additionally, our patient lacked typical psychological signs of an eating disorder, such as distorted body image perception or fear of weight gain. Moreover, the vomiting resulted from IBD-related complications rather than self-induced behaviour. Early recognition of IBD symptoms is critical for timely referral to the appropriate diagnostic process, minimizing delays and complications. As far as concern psychosocial comorbidities in feeding and eating disorders, in the last decade, research has documented a higher presence of comorbid autistic symptoms among people with eating disorders, especially in patients with anorexia nervosa [8]. Our case #3 emphasizes how, despite the manifest aberrant eating pattern in a boy affected by autism spectrum disorder, it is essential to investigate collateral symptoms not fully explained by the first diagnosis and to rule out other organic causes. Indeed, a concomitant neuropsychological disorder should not be exempt from performing additional investigations, especially in case of red flags. In our series, patient #3 presented with alternating bowel habits. Despite this feature being possible in patients with eating disorders, constipation is much more frequent. In contrast, diarrhoea, mostly when bloody, should always prompt further investigations to rule out organic causes. In this case, familial adenomatous polyposis was diagnosed. However, paediatricians should consider several common conditions, such as the abovementioned IBD, in the differential diagnosis.

A classical hallmark of eating disorders is the fear of gaining weight [9]. In patient #4, her fear of gaining weight was attributed to her family history (her father’s diabetes) rather than to an eating disorder. Even when more specific features of eating disorders are present, physicians should thoroughly consider personal and family history. TINU syndrome is prone to misdiagnosis because kidney damage may not occur simultaneously with uveitis; furthermore, tubulointerstitial nephritis may be silent or manifest itself with a subtle feature for weeks or months [10]. Initially, her symptoms (including fatigue, nausea, vomiting, and weight loss) were misdiagnosed as an eating disorder. However, neither psychotherapy nor enteral nutrition led to any improvement. Indeed, her symptoms worsened, along with unfavourable changes in laboratory indicators, after starting intravenous rehydration.

This finding emphasizes the importance of promptly considering alternative diagnoses when standard treatments for eating disorders are ineffective or worsen symptoms; ideally, before complications arise.

Feeding and eating disorders have a high intrafamilial risk [11]. Therefore, when screening for these disorders, a thorough investigation of family history is crucial, as highlighted by relevant interview items [12]. However, as emphasized by case #5, a collateral familial history revealing abnormal eating-related behaviours or other psychological comorbidities should not be exempt from considering serious medical causes of weight loss for which diagnostic delay may be fatal, such as GI tumours. In literature, the trichobezoar has also been reported as an anecdotal medical cause mimicking AN, as occurred in our case #6. Trichobezoars are a collection of hair or hair-like fibres in the GI tract, primarily in the stomach [13]. The initial diagnosis of this girl was an eating disorder; nevertheless, her mother had revealed trichotillomania in her daughter's history. Trichotillomania is frequently observed in young women with underlying psychiatric or behavioural issues. Common presenting symptoms of trichobezoar include abdominal pain, nausea, and vomiting, whereas weight loss is rarely observed [14]. Either CT scan or US can be diagnostic, whereas upper GI endoscopy can also be used as a diagnostic and therapeutic tool. Sometimes, however, trichobezoars are too large to be removed without surgical intervention.

Furthermore, paediatricians should also think about the endocrine causes of weight loss when approaching a patient with a suspected eating disorder. Addison’s disease, which may present with weight loss, apathy, and anxiety, may be mistaken for a psychiatric disorder, as highlighted by our case #8. Indeed, anorexia nervosa and Addison’s disease both present with similar clinical features. However, some different characteristics can help differentiate the two conditions: the most relevant include the absence of distorted self-perception and caloric restriction in Addison’s disease, characterized by typical electrolyte disturbances (hyponatremia and hyperkalaemia) as reported by Nicholls et al. [15]. Besides the diseases in our case series, other medical conditions can mimic eating disorders and lead to misdiagnosis as anorexia nervosa [16]. Among GI disorders, oesophageal achalasia should be considered in the differential diagnosis of cases with restricted oral intake, swallowing difficulty, and overt dysphagia [17]. This misdiagnosis has been reported both in children and adolescents [18, 19]. Eosinophilic esophagitis, another oesophageal disorder, can mimic an eating disorder and is increasingly common among children and adolescents [20]. Similarly, anorexia and malnutrition can be the presenting features of celiac disease due to malabsorption; given its high prevalence, it is pivotal to perform screening with total immunoglobulin A (IgA) and IgA antibodies directed against tissue transglutaminase [21].

Signs and symptoms of eating disorders can also mimic other endocrinopathies, beyond Addison’s disease: diabetes and thyroid disease need to be ruled out [22]. Interestingly, as for GI conditions, eating and endocrine disorders can also coexist or the first could develop as a complication of the latter one; indeed, in cases of undetected hypothyroidism, sinus bradycardia persisted despite weight gain [23].

Also, several chronic infections, such as tuberculosis, HIV, and giardiasis, can be misdiagnosed as eating disorders in children and adolescents [24]. The most common medical conditions that can present with signs and symptoms evocative of an eating disorder are summarized in Table 1.

**Table 1:** Most common differential diagnoses between medical condition and eating disorders in children *(modified from Hornberger LL *et al.,* 2021)*

*Gastrointestinal diseases*	
IBD	
Celiac disease	
Eosinophilic esophagitis	
Gastroesophageal reflux disease	
Achalasia	
Pancreatitis	
*Endocrine disorders*	
Addison’s disease	
Diabetes	
Thyroid disorder	
Hypopituitarism	
*Infections*	
Chronic infections (HIV, tuberculosis, etc.)	
Intestinal parasitosis (i.e. giardiasis)	
*Malignancies*	
Brain tumours	
Gastrointestinal tumours	
Other cancers	
*Others*	
Trichobezoar	

*IBD* inflammatory bowel disease, *HIV* human immunodeficiency infection

Moreover, particular attention should be directed towards cases of treatment-resistant anorexia nervosa (TR-AN)**.** An increasing body of evidence highlights the necessity of a thorough differential diagnosis prior to establishing a diagnosis of TR-AN [25]. Specifically, it is essential to exclude underlying organic or medical conditions that may mimic or contribute to the clinical manifestation of anorexia. This concern has been underscored in clinical reports where standard interventions—such as enteral nutrition via nasogastric tube and/or fluid resuscitation—failed to result in clinical improvement, or in some cases, led to further deterioration. Treatment resistance in suspected anorexia nervosa should thus be regarded as a diagnostic red flag, warranting careful re-evaluation and reassessment to rule out somatic conditions that could be misdiagnosed as an eating disorder [26].

When evaluating a child or adolescent with weight loss and suspected feeding or eating disorders, it is crucial to explore a variety of potential diagnoses. This assessment involves a thorough medical history, comprehensive physical exam, endocrine evaluation, and, if necessary, neuroimaging, especially when symptoms like headache and vomiting intermittently occur. The comprehensive evaluation should prioritize confirming whether weight concerns serve as the primary factor behind weight loss while also excluding other potential medical causes. When investigating thoroughly the medical history, it is crucial to appraise psychological aspects (i.e. body dysmorphism, fear of gaining weight, etc.) which are highly specific of eating disorders but absent in the other medical conditions. To help clinicians, web resources are published in multiple languages by the Academy for Eating Disorders [27]. Collateral history obtained from a parent may uncover disordered eating behaviours that the child or adolescent has denied or minimized.

Additionally, establishing a rigorous follow-up for these patients is essential. Healthcare providers should be aware to reassess the initial diagnosis if new symptoms emerge or if there is an inadequate response to conventional therapies.

## Strength and limits

The strengths of our work include the comprehensive literature review accompanying different educational clinical cases from a tertiary-level paediatric hospital mistakenly diagnosed as anorexia nervosa. The main limitations include our inability to report systematically data from all the patients with a similar clinical scenario who were admitted to our hospital.

## What is already known on this subject?

When approaching anorexia nervosa in paediatrics, several organic differentials must be taken into consideration.

## What this study adds?

Our case series underscores the significance of comprehensive history-taking and detailed physical examinations in diagnosing feeding and eating disorders and provides a synopsis of the most commonly organic diseases mistakenly diagnosed for anorexia nervosa from infancy to adolescence.

## Data Availability

No datasets were generated or analysed during the current study.
